# Strategies for enhancing social skills of individuals with intellectual disability: A systematic review

**DOI:** 10.3389/fresc.2022.968314

**Published:** 2022-09-13

**Authors:** Udeme Samuel Jacob, Isioma Sitamalife Edozie, Jace Pillay

**Affiliations:** ^1^ South African Research Chair in Education and Care in Childhood, Faculty of Education, University of Johannesburg, Johannesburg, South Africa; ^2^ Department of Special Education, Faculty of Education, University of Ibadan, Ibadan, Nigeria; ^3^ Department of Adult and Non - Formal Education, School of Early Childhood Care, Primary, Adult and Non -Formal Education, Federal College of Education (Technical) Asaba, Asaba, Nigeria

**Keywords:** social skills, intellectual disability (ID), intervention, strategies, treatment

## Abstract

Individuals with intellectual disability who suffer from comorbid mental health problems are likely to experience difficulties in socialising. Deficits in social skills are also associated with challenging behaviours and self-injury. This paper presents global evidence from a systematic review of literature on such issues as ‘interventions’; ‘social skills development’, and ‘individuals with intellectual disability’. A thorough search of various bibliographic databases identified 1 124 academic papers. Ten papers met the inclusion criteria for in-depth analysis concerning the use of interventions to develop social skills among individuals with intellectual disability. The study revealed that the social skills of individuals with intellectual disability had been fostered using different strategies, such as classroom-based intervention, emotional intelligence training, use of a peer network intervention, computer games of emotion regulation, and puppet play therapy. Furthermore, the findings suggest that various aspects like communication, bridging the gap in social skills deficits, emotional recognition and regulation, and adaptive behaviour were fostered using the identified intervention strategy. This review revealed that social skills interventions appeared modestly effective but may not be generalisable to school settings or self-reported social behaviour for individuals with intellectual disability. It is also necessary to increase the sample size in future studies to draw generalisable conclusions.

## Introduction

Various definitions of the term intellectual disability have been applied by different disciplines based on their perception of the condition. Early definitions emphasise biological and medical criteria, whereas educational criteria become more prominent once more teachers and psychologists became interested in the subject (1). The American Association on Intellectual and Developmental Disabilities (AAIDD) provided a widely recognised definition. Additional explanations have been provided in the *Diagnostic and Statistical Manual of Mental Disorders* (5th ed, DSM-5) of the American Psychological Association (APA) and the International Statistical Classification of Diseases and Related Health Problems (ICD-10) of the World Health Organization. According to the AAIDD (2), intellectual disability is characterised by significant limitations in intellectual functioning, and the conceptual, social, and practical skills that make up adaptive behaviour during developmental age (3, 4).

A delay in brain development during the developmental period severely restricts academic, social, emotional, and behavioural adjustments of individuals with intellectual disability (5, 6). In this regard, the condition indicates an impairment of mental and cognitive development, resulting in the inability of the individual to develop the adaptive skills required to cope with daily life. People with adaptive behaviour possess conceptual, social, and practical skills that enable them to function in their everyday lives (2). The implications are that individuals with intellectual disability who suffer from deficits in social skills are likely to have difficulty interpreting social signals in their interactions, increasing the likelihood of them being manipulated by others (7). Moreover, they may experience difficulties regulating their emotions when interacting with their peers (8, 9).

As opposed to the AAIDD, in the DSM-5 (APA), (10) social skills deficit among individuals with intellectual is described as adaptive functioning to rather than adaptive behaviour in the definition. It clarifies that adaptive functioning comprises three domains (conceptual, social, and practical) that determine an individual’s ability to cope with daily tasks (10). However, Salami (11) describes it as a developmental disorder that impairs an individual’s cognitive ability, resulting in a defect in the ability to learn and understand concepts. Individuals and children with intellectual disabilities can be found across the globe. According to the APA (10), the population of each country or region varies based on assessment and diagnosis. Based on Adebisi et al. (12), the prevalence of intellectual disabilities cuts across races, colours, socioeconomic status, and conditions in different countries.

These definitions of disabilities refer to impairments relating to conceptual, social, and practical skills that make it possible to carry out basic tasks. It also includes impairments related to mental abilities. The most significant characteristic of intellectual disability on an individual is a lack of social skills (13). In addition, individuals with intellectual disability may show a lack of social reciprocity, poor eye contact and facial expressions, a lack of nonverbal behaviours and gestures, and difficulty maintaining relationships with peers (14–16). Social skills are frequently challenging to demonstrate across contexts for individuals with intellectual disability. Katz and Lazcano-Ponce (17) maintain that a disability has profound social consequences for the affected individual.

There is evidence that comorbid mental health difficulties are associated with social challenges and weaker social skills among individuals with autism (18). Social deficits are also associated with challenging behaviours and self-injury in children and adolescents with autism spectrum disorder (ASD) (19). Compared to adults with intellectual disability only, challenging behaviours among adults with ASD and intellectual disability are four times higher (20). Depressive symptoms were negatively associated with self-reported friendship quality in adolescents with ASD without intellectual disability (21). Self-perceived social competence and depression symptoms were significantly correlated (22). Promoting social skills among individuals with intellectual disability may likely reduce challenging behaviours, address severe behavioural issues, and improve mental health.

Impairment in social skills functioning is the defining feature among individuals with intellectual, ASD and the combination of intellectual disability with ASD due to significant limitations in intellectual functioning (23). Such deficits significantly impact academic, adaptive, and psychological functioning (24). It is also associated with more severe intellectual disability and communication problems on both the verbal and nonverbal levels (25). Consequently, individuals may experience isolation in social situations (26), lower levels of acceptance by peers and teachers (27, 28), and significant social disadvantages (29). Poor social skills development is linked to this difference in the ability to form friendships (30). Individuals with intellectual disability usually encounter difficulties in making and maintaining friendships. It is common for their friendships to be characterised by a lack of warmth, closeness, and reciprocity in comparison to their peers, who are typically developing (30).

For people with disabilities to be successful in the workplace, they must be able to interact socially with others (31). People with mild or moderate intellectual disability generally have difficulty understanding directions, socialising, making choices, and demonstrating flexibility. However, a high IQ (well above the norm) is not necessarily indicative of a high quality of life and satisfaction (32, 33). In psychology, there has been a growing interest in other factors that determine how people function in life. Social nature is inherent in these factors. Therefore, social intelligence, interpersonal skills, emotional intelligence, and emotional competence have become increasingly popular since the early 1990s. It is also important to note that social skills are included in this group (34). The concept of social skills is complex and closely linked to the level of human functioning in society. A social function can only be achieved when individuals possess these essential skills (35, 36). It applies to individuals with intellectual norms and individuals with intellectual disability. Both groups live in the different social spaces of family, and local community or society.

However, there appears to be a disconnect between practitioners’ views of social skills and what they provide to students with disabilities in secondary schools (37). An individual’s social skills can be defined as “determinants that influence their ability to function effectively in social situations” (34, p. 5). There is a direct correlation between social skills and social training, which is also influenced by other factors. The concept of skills can be treated in singular or plural, made up of several components, for example, assertiveness, empathy skills, or building emotional bonds. Alternatively, specific social skills of a collection of individuals may be referred to as a collection of social skills in the plural. Social skills are related to social and emotional intelligence. Both types of intelligence can be considered the basis for developing the human ability to live in society. Understanding and managing people is part of this process (38).

Social skills also refer to the ability to understand and experience one’s own emotions as well as the emotions of others, which constitutes emotional intelligence. The term was introduced in 1990 by Salovey and Mayer (39). It is also important to note that emotions are the basis for both social and emotional types of intelligence. It is essential to use this approach to diagnose individuals with intellectual disability who are capable of empathising and do not lack feelings. Consequently, we can study the social and emotional intelligence of individuals with intellectual disability. Social impairments may also be more pronounced in individuals with intellectual disability during adolescence when social demands outweigh social skills (40), highlighting the need for interventions to enhance social competence.

Therefore, social skills deficits are an essential target for intervention (24, 41, 42). The development of effective instructional strategies for teaching social skills to individuals with intellectual disability and intellectual disability combined with ASD has received considerable attention over the past four decades (31, 43). This shows that evidence-based interventions for developing social skills often benefit individuals with intellectual disability. Despite this, less emphasis is placed on instructing them on skills they perceive as essential (44), such as asking for more specific instructions, responding appropriately to constructive criticism, and seeking assistance when necessary

It is important to note that previous reviews on strategies for fostering social skills have focused on studies that used video-based intervention for individuals with developmental disabilities, including ASD. Ayres and Langone (45) reviewed the use of video modelling to teach social and functional skills. In their study, they concluded that the use of video modelling for teaching these skills was highly effective. By categorising video modelling studies into adult, peer, self, and point of view models (46), presented an overview of video modelling research. Regardless of the type of model used, video modelling was effective. In Delano (47), the effectiveness of video modelling was reviewed, as well as the most used type of video modelling and the skill areas taught in ASD classes. Individuals with autism spectrum disorders are often taught social-communicative behaviours through video modelling (e.g., social initiation, verbal statements about play).

However, this review widened the scope because the focus was on an in-depth understanding of various interventions used for social skills development among individuals with intellectual disability. We formulated three research questions:

1. What are the characteristics of participants with intellectual disability?
2. What is the type and density of intervention?
3. What conclusions did the research studies draw?

## Methods

### Literature search

**Figure 1:**
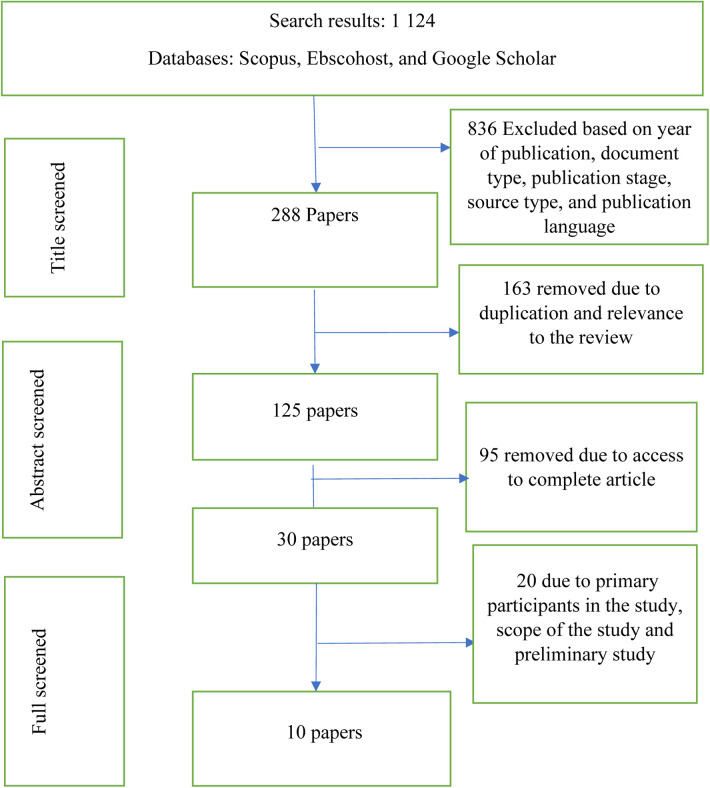
Flowchart of the systematic literature search.

Online electronic searches were conducted on the EBSCO Host, Scopus, EMBASE, PsycINFO and CINAHL databases. Eligibility criteria included social skills and intellectual disability subject heading. Key terms included ‘social skills’, ‘interventions’, ‘intellectual disability’ and the language of the publication needed to be English. In total 1,124 documents were found. A manual search of article reference lists was also performed (Figure 1). The reference lists of studies included in the electronic search were screened to identify additional studies.

### Selection criteria

The Preferred Reporting Items for Systematic Reviews and Meta-Analyses (PRISMA) determined the screening criteria (48, 49). We searched the literature on interventions designed to improve the social skills of individuals with intellectual disability. We did not limit the search to any location. The exclusion criterion was supported by the assertion that the systematic literature review conclusion was not influenced by the exclusion or inclusion of dissertations (50). Atlas.ti 22 was then used to screen and analyse the articles. Based on the three research questions raised, each author independently screened titles and abstracts for all articles:

In the data extraction, the selection stages and attributes were as follows:

1. It must have been published in English in an open access, peer-reviewed journal between January 2001 and February 2022, which was the end date for the most recent review (51).
2. Study participants must have been identified as individuals with intellectual disability or related conditions (48).
3. An empirical design must have been used (i.e., a single subject, experimental or quasi-experimental design). Qualitative and descriptive studies were excluded (52).
4. We only included articles that specifically addressed intellectual disability. We generally excluded articles that did not explicitly mention intellectual disability.
5. Our analysis only included articles related to interventions for enhancing social skills development.

### Outcome of search

Using the search terms ‘intellectual disability’, ‘social skills’, and ‘intervention’, 1,124 publications were found (PubMed, Scopus, Google Scholar, and Ebsco) (see Figure 1). We examined the records using the inclusion and exclusion criteria we established before the search. We removed 836 of the 1,124 articles due to their type, publication stage, source type, and language of publication. The remaining 288 articles were further screened for duplication and relevance to the review, leading to the removal of 163 more articles. Based on access to complete articles, 95 were removed, leaving 125 titles. The full text of the remaining 30 articles was read to get the perspectives of the authors resulting in the removal of 20 articles that did not meet the inclusion criteria (53). The remaining 10 studies were eligible for full-text review. In essence, only original research articles were included, indicating that conferences and review papers were excluded.

### Quality assessment

The quality of the review was ensured by searching duplicate articles. After evaluating each article based on the inclusion and exclusion criteria, 10 articles were selected. To ensure reliability, the authors independently coded a random sample of 20% of the included studies. The authors calculated inter-rater reliability using a percentage agreement and found that all codes agreed 87.3% of the time. Figure 1 shows the literature included and excluded at each stage.

### Ethics

The study did not require special ethical considerations because all the articles used for the review were freely available in the public domain.

### Data extraction

Ten articles were double coded by the same two blind raters on various demographic variables and outcome data on social competence (see Tables 1–4 for a complete list of articles included in the review). The measures coded included types of intervention, participants, number of participants, duration of studies, number of participants (male and female), intervention description, intervention density, study type/design, the statistical tool used and results by outcome (pre and post). Researchers consulted two colleagues from universities in South Africa to validate the information gathered: one from the Department of Early Childhood Education; and the other from the Department of Educational Psychology. All the identified studies were assessed and critiqued by the two colleagues.

**Table 1:** Included studies and participant characteristics.

Authors	Types of intervention	Participants	Number of participants	Duration of studies
Adeniyi and Omigbodun (54)	Classroom-based intervention	Pupils with intellectual disability	30	8 weeks
Adibsereshki et al. (55)	Emotional intelligence training	Students with intellectual disability	32	22 sessions (5 weeks)
Olçay Gül (56)	Combined use of video modeling and social stories	Individuals with intellectual disability	4	Not specified
Jacob et al. (57)	Peer tutoring and storytelling	Pupils with mild intellectual disability	34	30 sessions (10 weeks)
Kalyveza et al. (58)	Use of a peer network intervention	Adolescents with ASD	3	One to two sessions were run per week (6 months)
Kashani-Vahid et al. (59)	Computer games of emotion regulation	Children with intellectual disability	20	Not specified
Khodabakhshi-Kooalee et al. (60)	Puppets play therapy	boy children with intellectual disability	30	Eight sessions (25 min for each session, twice a week)
Park et al. (61)	Video modelling	Youth with intellectual disability	3	Each intervention session was implemented 1 or 2 days per week for approximately 15 min; each session involved five trials.
Plavnick et al. (62)	School-based social skills training	Adolescents with ASD and intellectual disability	4	4 or 5 days per week for 40 min each session
Olsson et al. (63)	Social skills training	Children and adolescents with ASD	296	Children were trained for 60 min and adolescents for 90 min per week (12 sessions)

**Table 2:** Participant gender, treatment and mean age.

Authors	No (male, female)	N (T, C)	Mean age ± SD (range)	Mean ± SD skill level/ IQ (pre-treatment)
Adeniyi and Omigbodun (54)	30 (16, 14)	30 (30, 0)	15.70 ± 1.89 (12–19)	19.5 ± 7.9
Adibsereshki et al. (55)	32 (0, 32)	32 (16, 16)	14–18	(IQ of 58–70).
Olçay Gül (56)	4	4 (4, 0)	20–25	35–55
Jacob et al. (57)	34 (16, 18)	32 (21, 13)	11.7	427.21; *η*^2^ = 0.799
Kalyveza (58)	3 (3, 0)	4 (4, 0)	13–14	Not specified
Kashani-Vahid et al. (59)	20	20 (10,10)	Not specified	50–70
Khodabakhshi-Kooalee et al. (60)	30 (30, 0)	30 (15, 15)	9–11	Not specified
Park et al. (61)	3 (1, 2)	3 (3, 0)	19	Not specified
Plavnick et al. (62)	4 (3, 1)	4 (4, 0)	14–17	Not specified
Olsson et al. (63)	296 (208, 88)	296 (296, 0)	7–17	>70

**Table 3:** Intervention description, intervention density, and total hours of intervention for behaviour analytic intervention studies.

Author and year of publication of original study (references)	Intervention	Intervention description	Intervention density	Total hours of treatment	Agent
Adeniyi and Omigbodun (54)	Role play	Participants received lessons in their classrooms from the adapted Explore curriculum. Each session consisted of an introduction of the topic of discussion, a self-talk story, where the teacher gave a narrative overview, and role-plays.	45 min/lesson; 3–4 times a week for 8 weeks	18–24 h	Teachers
Adibsereshki et al. (55)	EI training	The experimental groups had 22 sessions of EI training, while the control group did not have any; during the intervention, they only had their regular school programme. The pretest was carried out for the two groups (experimental and control) before the intervention started, and the posttest was done after the intervention.	22 sessions of 45 min of instruction.	16 h	Not specified
Olçay Gül (56)	Video intervention	Probe sessions were carried out immediately before participants were allowed to perform the skill. Seven steps were followed in the intervention sessions: (a) watch the video before entering the setting where the target skill was to be exhibited; (b) watch the video in a designated classroom (in silence and without interruption); (c) provide an attentional cue; (d) verbally reinforce the participant with attention-directing behaviour; (e) watch video; (f) verbally reinforce the video-watching behaviour of the individual; and (g) direct the participant to the setting where the behaviour is to be exhibited.	Not specified	Not specified	The researcher, observers and stimuli presenters
Jacob et al. (57)	Peer tutoring/storytelling	The treatment package began with a pretest exercise. The treatment lasted for 10 weeks, with 3 weekly sessions of 50 min each. Posttests were conducted at the end of the 10 weeks to assess the programme’s effectiveness.	The treatment lasted for 10 weeks, with 3 weekly sessions of 50 min each.		Research assistant
Kalyveza (58)	Individualised intervention/circle time	The study used a manual programme called the circle. Participants had to complete five steps for each activity during each session.	1 h/session	Not specified	Peer partners
Kashani-Vahid et al. (59)	Computer game	The “EmoGalaxy” game, designed and developed by the Robotics and Artificial Intelligence of University of Tehran, takes place in a system composed of four planets. Each planet refers to one of the primary emotions. These four feelings are happiness, sadness, fear, and anger. There are several buildings on each planet and clicking on each one will open a game.	15 sessions (45 min) for one and a half months (45 days)	11 h	Not specified
Khodabakhshi-Kooalee et al. (60)	Puppet play therapy	Three puppets were used in the study:
		• a puppet of a boy (representing truth) • a puppet of a crow (representing a mistake) • a puppet of a grandmother (representing grandmother) Role-playing sessions included the following:
		1. Understanding the different types of emotions such as sadness and happiness. 2. Enhancing verbal and nonverbal skills, such as speaking, listening, asking questions, expressing emotions, and expressing social compliments. 3. Awareness of personal rights and respect for others’ rights. 4. Recognising money and its counting and how to go shopping; and 5. Using a variety of phones and public transport.	The intervention group received puppet play therapy in eight sessions (25 min for each session, twice a week)	3 h 33 min	Not specified
Park et al. (61)	Video modelling	A researcher taught each participant using video modelling. At the beginning of each session, the researcher reminded the participants that they would be practising situations they would encounter at work. Afterwards, the researcher placed an iPad on the table and instructed each student to watch a video. Additionally, the researcher acted as a model and showed demonstrations of target skills in the video. The researcher explained to the student that they would practise the skill after watching the video.	1–2 days per week, 15 min per session; 5 trials per session		Not specified
Plavnick et al. (62)	Video-based group instruction (VGI)	The study used 18 videos to teach six behaviours; three versions of each were developed with different models, stimuli, and language used in each video to promote variability in response. Moreover, each video included relevant antecedents, the target behaviour, and naturally occurring consequences that would serve as potential tangible reinforcers.	The VGI sessions were conducted four or 5 days per week for 40 min each.	Not specified	Teachers
Olsson et al. (63)	Social skills group training (SSGT) for children and adolescents	A total of 50 clinicians delivered the intervention (39 psychologists, 5 social workers, 3 nurses, 2 special educators, and one speech-language therapist) with an average of 6 years (range, 1–36 years) clinical experience in autism. They received systematic training in the programme, which included classroom instruction, supervision, and feedback on recorded sessions. A continuous supervisory process was implemented following training to ensure the integrity of the SSGT. Using a checklist containing 11 items regarding protocol adherence and trainer skills in implementing the basic principles of the SSGT during monthly trainer meetings (e.g., positive reinforcement, modelling, prompting), a random sample of 27 (25%) video-recorded sessions was assessed. There were three levels of adherence: zero for no adherence; one for some adherence; and two for full adherence. A mean score was computed.	Children were trained for 60 min and adolescents for 90 min per week in groups of 4 to 8 participants by 2 trainers. In this study, 12 sessions of increasing complexity were fully standardised. Participants were randomly assigned to 12-week SSGT	Not specified	Medical practitioners

**Table 4:** Research design, sampling technique, sample size and outcome in the included studies.

First author and year of publication of original study (references)	Number of participants	Study type/Design	Method of data analysis used	Results by outcome (pre and post)
Adeniyi and Omigbodun (54)	30	Non-experimental	MANOVA	In the post-intervention analysis, there was a 20% reduction in participants with severe social skills impairments.
				The number of participants with minimal or no social skills increased by 13.3%.
Adibsereshki et al. (55)	32	Quasi-experimental involving a pretest, posttest design and control group	ANOVA	Experimental and control groups scored significantly differently after the intervention program.
				Communication scores were higher in the experimental group posttest and the follow-up for the experimental group.
Olçay Gül (56)	4	Multiple probe design	Descriptive analysis through the subjective evaluation approach	Participants acquired all the target social skills correctly. Participants maintained these skills over time and generalised them across settings, situations, and individuals.
				Moreover, the social validity data collected through semistructured interviews were generally positive.
Jacob et al. (57)	34	Quasi-experimental pretest-posttest and control group research design with a 3 × 2 factorial matrix	ANCOVA	There was a significant difference in the pretest and posttest results among the three groups.
				In addition to the effect of gender on participants’ social skills the interaction effect of treatment and gender was also significant.
Kalyveza et al. (58)	3	A single-case-multiple-baseline design across participants with probe assessments in every phase of the study (intervention and maintenance)	Range and percentage	All three participants’ social skills improved in an unstructured environment—namely, the playground—during recess, and in unfamiliar terrain they visited during school excursions.
				Researchers found that children with autism benefitted from individualised interventions that helped them acquire new social skills and generalise them.
Kashani-Vahid et al. (59)	20	This quasi-experimental study involved two groups and random placement with pretest and posttest.	ANCOVA	Cognitive computer games significantly increased social skills scores and their components in children. Computer cognitive games positively affected all aspects of children’s social skills. Test group scores before and after the test showed significant differences.
				In the posttest, there was a significant difference between the mean scores in social skills and their components.
Khodabakhshi-Kooalee et al. (60)	30	A quasi-experimental study with pre/posttest design		There were significant differences in the mean scores of six subscales of adaptive behaviour, including violent and disruptive, antisocial, rebellious, untrustworthy, stereotyped, unacceptable eccentric, and Vineland’s social maturity after the intervention.
				Furthermore, findings did not show significant differences between five subscales of adaptive behaviour, including withdrawal, inappropriate social, unacceptable vocal habits, hyperactive tendencies, and psychological disturbance.
Park et al. (61)	3	A multiple probe design across behaviours	Visual analysis, median and split-middle method	Despite making significant progress in acquiring targeted skills between baseline and intervention, all participants struggled with generalising their responses.
				A functional relation existed between the intervention and the dependent variable.
				In the second trial, all three students displayed all behaviours except one (accuracy in one of two trials) for up to two weeks after the last intervention.
Plavnick et al. (62)	4	This study utilised a multiple probe design across social skills domains (i.e., paired behaviours) to assess the effects of VGI on participants’ social behaviour.	Percentage and frequency	Three out of four participants demonstrated successful outcomes with VGI regarding teaching novel social behaviour, while the fourth participant showed mixed results. The effects of long-term maintenance were observed for two participants, but generalisation results were inconclusive.
				Based on the results, some adolescents with ASD-ID may benefit from using VGI within high school curricula.
Olsson et al. (63)	296	Randomised controlled trial	R software version 3.2 and IBM SPSS statistics version 24.	A significant treatment effect was observed only for parental ratings for the adolescent subgroup.
				Adaptive functioning and clinical severity were also moderately impacted by treatment.

## Results

This study focuses on a systematic literature review on the social skills of students with intelligent disability.

This study reviewed ten articles.

### Types of intervention adopted in the studies

The 10 articles included in the review adopted 12 different interventions for fostering the social skills of individuals with intellectual disability. The interventions were classroom-based interventions (54), emotional intelligence training (55), and combined use of video modelling and social stories (56). Other interventions used were peer tutoring and storytelling (57), the use of a peer network intervention (58), and computer games of emotion regulation (59). The studies of Khodabakhshi-Kooalee et al. (60) and Park et al. (61) investigated puppet play therapy and video modelling, respectively. The review also examined the significance of school-based social skills training (62) and social skills training (63). Two studies in the review used two interventions to foster social skills among individuals with intellectual disability. The study combined video modelling and social stories (56) and peer tutoring and storytelling (57) to enhance social skills.

### Participants included in the study

Table 1 revealed there were four hundred fifty-six (456) participants across the 10 studies included in this review. The study by Olsson et al. (63) had the highest number of participants at 296, or 64.9% of the study participants. Kalyveza et al. (58) and Park et al. (61) had the least number of participants at 3. Adeniyi and Omigbodun (54) and Khodabakhshi-Kooalee et al. (60) had 30 participants in each study, which was 13.2% of the total participants. The study of Jacob et al. (57) had 34 participants, which made up 7.5% of the participants.

### Duration of intervention

Two of the studies included did not specify the duration of the interventions. The study conducted by Kalyveza et al. (58) had one to two sessions per week over 6 months, which was the longest period of study. The study by Olsson et al. (63) varied the duration of the intervention with children being trained for 60 min and adolescents for 90 min per week over 12 sessions. Two studies did not specify the number of weeks or months the interventions lasted (56, 59). Adeniyi and Omigbodun (54) provided an 8-week intervention, while Jacob et al. (57) provided 30 sessions over a period of 10 weeks. Furthermore, Plavnick et al. (62) provided interventions four to five times weekly with each session lasting 40 min.

### Participant characteristics and treatment distribution

In Table 2 the characteristics of the participants of the included studies are presented. The review revealed that while both male and female participants were included in the studies, three of them used only one gender. The studies with both genders made up 60% of the included studies, while the remaining 40% did not have both genders as study participants. Adibsereshki et al. (55) used only female participants. Kalyveza (58) and Khodabakhshi-Kooalee (60) used only males as study participants. However, Khodabakhshi-Kooalee (60) stated that using only boys was a study limitation. Two of the studies, Olçay Gül (56) and Kashani-Vahid et al. (59), did not indicate the gender of the participants. The cumulative number of participants in the study included in this review were two hundred and seventy-seven (277), 61% of the participants were males, 34% (155) were females, and 5% (24) unidentified. The table also displays the mean age of the participants and the IQ mean. The mean age of the participants ranged from 7 to 25 years. Kashani-Vahid et al. (59) did not specify the age range of study participants.

### Intervention description, intervention density, and total hours of intervention for behaviour analytic intervention studies

Table 3 presents the different interventions engaged by various authors. Researchers and teachers were the primary agents in administering the treatment to the participants. As shown in Table 3, various interventions were employed. Generally, treatment agents (e.g., therapists) provided interventions in a single treatment session or multiple sessions per week (25–90 min per session). The treatment duration in the social skills training and support studies tended to be long, ranging from 3 to 24 weeks. As shown in the intervention description, different methods and curricula were used across the studies. Due to higher weekly treatment densities and more prolonged treatment durations, the total contact hours for fostering social skills training and support studies were higher. Total contact hours for each intervention type also differed.

### Study design

One of the studies included used a non-experimental research design. Three studies adopted a quasi-experimental design with pretest, posttest, and control groups, and two adopted a multiple probe design. In addition, each study adopted a single-case-multiple-baseline design and randomised controlled trial. It was evident from the data that 30% of the included studies used a quasi-experimental design with the pretest, posttest, and control groups, and 20% used a multiple probe design.

### Outcome measurement

There was an assessment of the quality and quantity of various aspects of social skills (for example, understanding, practical application, and satisfaction levels). Some studies evaluated the impact of the intervention on adaptive functioning and clinical severity, that is, the core deficits of ASD (63). Several studies assessed social skills (57, 59), communication (55), bridging the gap in social skills deficits (54), emotional recognition and regulation (59), and adaptive behaviour and social skills (60). Three studies had another perspective, such as agencies offering supported employment, and two studies sought to gather the view of parents of persons with intellectual disability. Four studies explored the perspectives of participants with intellectual disability, two sought teachers’ perspectives, and one explored the perspectives of parents and teachers of participants with intellectual disability. A study used observers to identify changes in the expected behaviour of participants with intellectual disability.

### The main finding of the studies

We located ten studies that were relevant to this review, with seven studies involving 157 individuals with intellectual disability (54–57, 59–61). In addition, one study involved 296 individuals with comorbid conditions (ASD with intellectual disability) (63), and one had three individuals with ASD as study participants. Table 3 provides descriptions of the intervention, techniques and density of each study, and the agent of the intervention. Table 4 describes the method of data analysis and outcomes for each social skills intervention study. Four of the ten studies were quasi-experimental research, three adopted multiple probes, and one adopted a non-experimental research design. Moreover, one study adopted a single-case-multiple-baseline design, while another used a randomised, pragmatic clinical trial.

A different level of significance was reported based on post-intervention analysis by Adeniyi and Omigbodun (54), that there was a 20% reduction in participants with severe social skills impairments. In addition to observing a 13.3% increase in participants with minimal or no social skills, Olsson et al. (63) found that only parental ratings for the adolescent subgroup showed a significant treatment effect. Treatment also moderately affected adaptive functioning and clinical severity. Furthermore, there was a considerable difference in the mean scores for six subscales of adaptive behaviour, including violent and disruptive, antisocial, rebellious, untrustworthy, stereotyped, unacceptable eccentric, and Vineland’s social maturity (60).

According to Kalyveza et al. (58), all three participants’ social skills improved in an unstructured environment, including on the playground, during recess, and in unfamiliar terrain during school excursions. Children with autism are also found to benefit from individualised interventions that help them to acquire and generalise social skills. Furthermore, findings did not show significant differences between five subscales of adaptive behaviour, including withdrawal, inappropriate social behaviour, unacceptable vocal habits, hyperactive tendencies, and psychological disturbance (60). On the other hand, Kashani-Vahid et al. (59) found that cognitive computer games improved children’s social skills scores and their components. This was true of all aspects of social skills. Score differences between groups before and after the test were significant.

A significant difference was found between the mean scores of social skills and their components in the posttest. Jacob et al. (57) reported that the pretest and posttest results of the two experimental and control groups were significantly different, and that gender substantially influenced the participants’ social skills. According to Olçay Gül (56), the intervention resulted in participants maintaining appropriate skills over time and generalising them across settings, situations, and individuals. Moreover, the social validity data collected through semistructured interviews were generally positive. However, Jacob et al. (57) concluded that the interaction effect of treatment and gender was also significant. Using a multiple probe design across several domains of social skills (i.e., paired behaviours), Plavnick et al. (62) evaluated the effects of virtual reality intervention on participants’ social behaviour. Three out of four participants reported successful outcomes in learning novel social behaviours, while the fourth participant showed mixed results. Two participants were observed to benefit from long-term maintenance, but generalisation was impossible. According to the study, using virtual reality intervention in high school curricula may help adolescents with ASD and intellectual disability.

## Discussion of findings

To our knowledge, this is the first systematic review evaluating the effectiveness of strategies for enhancing social skills among people with intellectual disability. Of the 1,224 studies identified, ten potentially relevant studies met the predetermined inclusion criteria. There were overarching aims to enhance social skills. Social skills intervention groups differed in structure, contents, and duration between the studies. Moreover, there was no single outcome measure used in the studies. According to the studies, a relatively consistent terminology was used to refer to intellectual disability. Almost all the research studies adopted descriptive and inferential statistics method of data analysis.

This systematic review did not have questions about the equality of systematised data since the findings from quantitative and qualitative studies were not combined. This was because it would have been challenging to reconcile the results of qualitative case studies with those of quantitative studies (54, 57). In addition, it appeared that the study that presented the most reliable design for making statements about the results of their intervention presented comparatively low but accurate results. The learners’ adaptive functioning improved significantly (63).

The studies investigated the effectiveness of social skills interventions in improving communication skills, bridging the gap in social skills deficits, adaptive behaviour, emotional recognition, and regulation. Furthermore, this finding provides valuable insight into participants’ self-awareness of their social competence. It is more consistent with reports from parents and teachers that individuals reported learning correct social skills but were not practising them in social settings. The results indicated that participants in the intervention did not feel they were improving their ability to perform social skills in real-life situations.

Moreover, the study that presented the most accurate results appeared to have presented comparatively low but accurate findings despite its reliable design. Although participants struggled to generalise their responses between baseline and intervention (61), significant improvements were observed in their acquisition of targeted skills. Moreover, there may be a consensus issue regarding the assessment of the effect on participants’ adaptive functioning as well as their clinical severity. Among the adolescent subgroup, Olsson et al. (63) found that only parental ratings were significantly influenced by treatment, as other outcomes were not explicit. The treatment moderately improved participants’ adaptive functioning and clinical severity (Payne et al., 1995). Participants’ social skills improved across different settings (56).

The issue of improving the social skills of participants in various situations emerged. There was a 20% reduction in participants with severe social skills impairments (54). According to Plavnick et al. (62), some adolescents with ASD-ID may benefit from VGI within the high school, but this may not be sufficient for generalization. In addition to the effect of gender on participants’ social skills, the interaction effect of treatment and gender was also significant (57). The conclusion of this review is consistent with that of Adibsereshki et al. (55), who determined significant differences between experimental and control groups following an intervention programme using a quasi-experimental design with a pretest, posttest, and control group research design. A higher communication score was found for the experimental group following the posttest and follow-up.

A significant limitation was the lack of a standard definition of social skills. Some social skills are universal (e.g., greetings, initiating conversations), while others are individualistic and sometimes represent complex behaviours (e.g., problem-solving skills, self-control). There is a lack of consensus regarding which behaviours fall within the social skills domain, which poses a challenge for scientific research. As a result, comparisons between studies are difficult. A second issue is that certain behaviours (such as self-control) are hard to operationalise and assess, making it difficult to evaluate the effectiveness of any treatment. Moreover, the sample sizes are insufficient for meaningful statistical analysis. As shown in Table 1, four studies had fewer than 10 participants (56, 58, 61, 62).

It is important to have larger samples to increase internal and external validity. There is a paucity of research examining the effects of maturation and time throughout treatment using group designs. Four studies used a comparative group design (55, 57, 59, 60). There were two studies with placebo controls (real randomised controlled studies) (57, 60). According to the findings of these two studies, interventions effectively improved the social skills of individuals with intellectual disability.

## Conclusion

We reviewed social skills interventions for people with intellectual disability or autism spectrum disorders with lower functioning delivered by different specialists. It is therefore not possible to directly compare our findings with existing reviews. However, there have been reviews of psychosocial interventions for children with autism and other neurodevelopmental disorders delivered by specialist providers. An extant review on the effectiveness of interventions for individuals with intellectual disability showed evidence that intervention is adequate. Our review thus complements the findings of other reviews and have strong relevance for improving the social skills of individuals with intellectual disability.

There are several avenues for future research. It is evident that more extensive scaled, more methodologically rigorous studies are necessary to: (a) determine whether group social skills interventions are associated with improved outcomes; and (b) identify the most effective methods for increasing skills acquisition within and beyond the group setting, as well as reducing secondary effects of social skills impairments. It is also necessary to conduct further research to determine whether the content of social skills interventions should differ according to participant characteristics, such as sex and age (e.g., due to gender differences in social skills development). In addition, there are few validated measures for assessing social skills in individuals with intellectual disabilities, both quantitatively and qualitatively. Normative thresholds should be established for existing measures (e.g., those used with younger or other clinical populations), or new measures should be developed and validated.

Our review has several limitations. Due to resource constraints, we only included English-language publications. Second, we adopted a reductionist approach: we excluded social skills intervention for persons with disability and articles that were not open access. While this was to maximise study homogeneity, an implication is that our review does not enable analyses of the potential mediating and moderating mechanisms that may be integral to intervention among individuals with other disabilities that are not necessarily intellectual disability. Thirdly, we excluded publications in press or books, so publication bias remains possible (e.g., omitting unpublished studies). It is also important to state that the number of studies included was limited.

## Data Availability

The original contributions presented in the study are included in the article.

## References

[1] Heward WL. Exceptional children: An introduction to special education. (8th ed.) Upper Saddle River, NJ: Merrill/Prentice-Hall (2003).

[2] American Association on Intellectual and Developmental Disabilities. Intellectual disability: defining criteria for intellectual disability. AAIDD (2022). Available at: https://www.aaidd.org/intellectual-disability/definition

[3] Eripek S. Zihinsel yetersizliği olan çocuklar [Children with intellectual disabilities]. Maya Akademi (2009).

[4] Hourcade J. Mental retardation. ERIC Digest (2002). Available at: https://eric.ed.gov/?id=ED473010

[5] Oyundoyin JO. Excluding the excluded: The ordeals of persons with special needs. Nigeria: A Faculty of Education Lecture delivered at the University of Ibadan (2013).

[6] Jacob US Oyefeso EO Adejola AO Pillay J. Social studies performance of pupils with intellectual disability: the effect of demonstration method and storytelling. Elem Educ Online. (2022) 21(1):36–47. www.ilkogretim-online.org/fullt¶ext/218-1644033097.pdf

[7] Horner-Johnson W Drum CE. Prevalence of maltreatment of people with intellectual disabilities: a review of recently published research. Ment Retard Dev Disabil. (2006) 12:57–69. 10.1002/mrdd.20097 16435331

[8] Baurain C Nader-Grosbois N. Socio-emotional regulation in children with intellectual disability and typically developing children in interactive contexts. Alter. (2012) 6(2):75–93. 10.1016/j.alter.2012.02.001

[9] Baurain C Nader-Grosbois N Dinnoe C. Socio-emotional regulation in children with intellectual disability and typically developing children, and teachers’ perceptions of their social adjustment. Res Dev Disabil. (2013) 34(9):2774–87. 10.1016/j.ridd.2013.03.022 23810924

[10] American Psychiatric Association. Intellectual disability. (2013). Available at: www.dsm5.org/Documents/Intellectual Disability Fact Sheet.pdf

[11] Salami GA. Stress among the caregivers of persons with intellectual disability in Nigeria. In: Fakolade OA Osisanya O Komolafe AF, editors. Dynamics of special education. Department of Special Education University of Ibadan (2019). p. 199–206.

[12] Adebisi RO Rasaki SA Liman AN. The prevalence, identification process and intervention strategies of children with intellectual disabilities: a report of an institution’s fieldwork. Int J Innov Educ Res. (2016) 4(1):54–61. 10.31686/ijier.vol4.iss1.509

[13] Matson JL Dempsey T LoVullo SV. Characteristics of social skills for adults with intellectual disability, autism and PDD-NOS. Res Autism Spectr Disord. (2009) 3(1):207–13. 10.1016/j.rasd.2008.05.006

[14] Hodges H Fealko C Soares N. Autism spectrum disorder: definition, epidemiology, causes, and clinical evaluation. Transl Pediatr. (2020) 9(Suppl 1):S55–65. 10.21037/tp.2019.09.09 32206584 PMC7082249

[15] Hartley SL Birgenheir D. Nonverbal social skills of adults with mild intellectual disability diagnosed with depression. J Ment Health Res Intellect Disabil. (2009) 2(1):11–28. 10.1080/19315860802601317 20046932 PMC2758786

[16] Marrus N Hall L. Intellectual disability and language disorder. Child Adolesc Psychiatr Clin N Am. (2017) 26(3):539–54. 10.1016/j.chc.2017.03.001 28577608 PMC5801738

[17] Katz G Lazcano-Ponce E. Intellectual disability: definition, etiological factors, classification, diagnosis, treatment and prognosis. Salud Publica Mex. (2008) 50(2):132–41. 10.1590/s0036-36342008000800005 18470340

[18] Ratcliffe B Wong M Dossetor D Hayes S. The association between social skills and mental health in school-aged children with autism spectrum disorder, with and without intellectual disability. J Autism Dev Disord. (2015) 45(8):2487–96. 10.1007/s10803-015-2411-z 25758822

[19] Waters P Healy O. Investigating the relationship between self-injurious behaviour, social deficits, and co-occurring behaviours in children and adolescents with autism spectrum disorder. Autism Res Treat. (2012) 2012:156481. 10.1155/2012/156481 23193469 PMC3502765

[20] McCarthy J Hemmings C Kravariti E Dworzynski K Holt G Bouras N Challenging behaviour and co-morbid psychopathology in adults with intellectual disability and autism spectrum disorders. Res Dev Disabil. (2010) 31(2):362–6. 10.1016/j.ridd.2009.10.009 19954927

[21] Whitehouse AJ Durkin K Jaquet E Ziatas K. Friendship, loneliness and depression in adolescents with asperger's syndrome. J Adolesc. (2009) 32(2):309–22. 10.1016/j.adolescence.2008.03.004 18692233

[22] Lee K. Predictors of depression in children with high-functioning autism spectrum disorders: the relationship between self-perceived social competence, intellectual ability, and depressive symptomology (UMI No. 3372072). [Doctoral dissertation, state university of New York at buffalo]. ProQuest Dissertations Publishing (2010).

[23] Srivastava AK Schwartz CE. Intellectual disability and autism spectrum disorders: causal genes and molecular mechanisms. Neurosci Biobeh Rev. (2014) 46(Pt 2):161–74. 10.1016/j.neubiorev.2014.02.015 PMC4185273 24709068

[24] Elliott SN Malecki CK Demaray MK. New directions in social skills assessment and intervention for elementary and middle school students. Exceptionality. (2001) 9(1–2):19–32. 10.1207/S15327035EX0912_3

[25] Dagnan D. Psychosocial interventions for people with intellectual disabilities and mental ill-health. Curr Opin Psychiatry. (2007) 20(5):456–60. 10.1097/YCO.0b013e3282ab9963 17762587

[26] Kampert AL Goreczny AJ. Community involvement and socialization among individuals with mental retardation. Res Dev Disabil. (2007) 28(3):278–86. 10.1016/j.ridd.2005.09.004 16837163

[27] Baker J. Contributions of teacher-child relationships to positive school adjustment during elementary school. J Sch Psychol. (2006) 44(3):211–29. 10.1016/j.jsp.2006.02.002

[28] Buyse E Verschueren K Doumen S Van Damme J Maes F. Classroom problem behavior and teacher-child relationships in kindergarten: the moderating role of classroom climate. J Sch Psychol. (2008) 46(4):367–91. 10.1016/j.jsp.2007.06.009 19083364

[29] Kozma A Mansell J Beadle-Brown J. Outcomes in different residential settings for people with intellectual disability: a systematic review. Am J Intellect Dev Disabil. (2009) 114(3):193–222. 10.1352/1944-7558-114.3.193 19374466

[30] Tipton LA Christensen L Blacher J. Friendship quality in adolescents with and without an intellectual disability. J Appl Res Intellect Disabil. (2013) 26(6):522–32. 10.1111/jar.12051 23620246

[31] Phillips BN Kaseroff AA Fleming AR Huck GE. Work-related social skills: definitions and interventions in public vocational rehabilitation. Rehabil Psychol. (2014) 59(4):386–98. 10.1037/rep0000011 25221959

[32] Campbell A. Subjective measures of well-being. Am Psychol. (1976) 31(2):117–24. 10.1037/0003-066X.31.2.117 1267244

[33] Herrnstein RJ Murray C. The bell curve: Intelligence and class structure in American life. Free Press (1994).

[34] Matczak A. Kwestionariusz kompetencji społecznych (Questionnaire of social competences). Warszaw: Pracownia testów Psychologicznych (2007).

[35] Argyle M. Nowe ustalenia treningu umiejętności społecznych (new social skills training findings). In: Domachowski W Argyle M, editors. Reguły życia społecznego. Oksfordzka psychologia społeczna. Warszaw: PWN (1994).

[36] Argyle M. Zdolności społeczne (social skills). In: Moscovici S, editors. Psychologia społeczna w relacji ja-inni. WSiP (1998).

[37] Agran M Hughes C Thoma CA Scott LA. Employment social skills: what skills are really valued? Career Dev Transit Except Individ. (2016) 39(2):111–20. 10.1177/2165143414546741

[38] Strelau J. Inteligencja człowieka (Human intelligence). Warszawa: Wydawnictwo Żak (1997).

[39] Mayer JD Salovey P Caruso DR Sitarenios G. Emotional intelligence as a standard intelligence. Emotion. (2001) 1(3):232–42. 10.1037/1528-3542.1.3.232 12934682

[40] Picci G Scherf KS. A two-hit model of autism: adolescence as the second hit. Clin Psychol Sci. (2015) 3(3):349–71. 10.1177/2167702614540646 26609500 PMC4655890

[41] Coie J Terry R Lenox K Lochman J Hyman C. Childhood peer rejection and aggression as predictors of stable patterns of adolescent disorder. Dev Psychopathol. (1995) 7(4):697–713. 10.1017/S0954579400006799

[42] Spence SH. Social skills training: Enhancing social competence with children and adolescents. Windsor: Nfer-Nelson (1995).

[43] Westling DL Fox L. Teaching students with severe disabilities. 3rd ed. Merrill Prentice-Hall (2004).

[44] Kasari C Shire S Factor R McCracken C. Psychosocial treatments for individuals with autism spectrum disorder across the lifespan: new developments and underlying mechanisms. Curr Psychiatry Rep. (2014) 16(11):512. 10.1007/s11920-014-0512-6 25248342

[45] Ayres KM Langone J. Intervention and instruction with video for students with autism: a review of the literature. Educ Train Autism Dev Disabil. (2005) 40:183–96.

[46] McCoy K Hermansen E. Video modeling for individuals with autism: a review of model types and effects. Educ Treat Child. (2007) 30(4):183–213. 10.1353/etc.2007.0029

[47] Delano ME. Video modeling interventions for individuals with autism. Remedial Spec Educ. (2007) 28(1):33–42. 10.1177/07419325070280010401

[48] Ghafari M Baigi V Cheraghi Z Doosti-Irani A. Correction: the prevalence of asymptomatic bacteriuria in Iranian pregnant women: a systematic review and meta-analysis. PLoS One. (2016) 11(10):e0165114. 10.1371/journal.pone.0165114 27336476 PMC4919037

[49] Jacob US Pillay J Oladipupo OO Eni-Olorunda T Asiru AB. Music therapy for individuals with intellectual disability: a systematic review. Int J Early Childhood Special Educ. (2022) 14(2):49–59. 10.9756/INT-JECSE/V14I2.006

[50] Vickers A Smith C. Incorporating data from dissertations in systematic reviews. Int J Technol Assess Health Care. (2000) 16(2):711–3. 10.1017/S0266462300101278 10932436

[51] Joseph LM Seery ME. Where is the phonics? A review of the literature on the use of phonetic analysis with students with mental retardation. Remedial Spec Educ. (2004) 25(2):88–94. 10.1177/07419325040250020301

[52] Wise JC Sevcik RA Romski M Morris RD. The relationship between phonological processing skills and word and nonword identification performance in children with mild intellectual disabilities. Res Dev Disabil. (2010) 31(6):1170–5. 10.1016/j.ridd.2010.08.004 20846821

[53] Kretzmann M Shih W Kasari C. Improving peer engagement of children with autism on the school playground: a randomized controlled trial. Behav Therapy. (2015) 46(1):20–8. 10.1016/j.beth.2014.03.006 25526832

[54] Adeniyi YC Omigbodun OO. Effect of a classroom-based intervention on the social skills of pupils with intellectual disability in southwest Nigeria. Child Adolesc Psychiatry Ment Health. (2016) 10(1):29. 10.1186/s13034-016-0118-3 27594901 PMC5010731

[55] Adibsereshki N Shaydaei M Movallali G. The effectiveness of emotional intelligence training on the adaptive behaviours of students with intellectual disability. Int J Dev Disabil. (2016) 62(4):245–52. 10.1179/2047387715Y.0000000014

[56] Olçay Gül S. The combined use of video modelling and social stories in teaching social skills for individuals with intellectual disability. Educ Sci: Theory Pract. (2016) 16(1):83–107. 10.12738/estp.2016.1.0046

[57] Jacob US Pillay J Ayandokun JC Oyundoyin JO. Social skills of pupils with mild intellectual disability: do peer tutoring, storytelling and gender play a role? Univ J Educ Res. (2021) 9(12):1887–97. 10.13189/ujer.2021.091201

[58] Kalyveza S Gkogkos G Maridaki-Kassotaki K Gena A Antonopoulou K. Promoting the social skills of adolescents with autism spectrum disorder (ASD) with the use of a peer network intervention. Learning Disabil: Contemporary J. (2020) 18(2):243–67. https://files.eric.ed.gov/fulltext/EJ1281044.pdf

[59] Kashani-Vahid L Mohajeri M Moradi H Irani A. Effectiveness of computer games of emotion regulation on social skills of children with intellectual disability. In: 2018 2nd National and 1st International Digital Games Research Conference: Trends, Technologies, and Applications (DGRC) (2018). p. 46–50.

[60] Khodabakhshi-Kooalee A Falsafinejad MR Rezaei S. Effectiveness puppet play therapy on adaptive behaviour and social skills in boy children with intellectual disability. Caspian J Pediatr. (2018) 4(1):271–7. http://caspianjp.ir/article-1-85-fa.pdf

[61] Park J Bouck EC Duenas A. Using video modeling to teach social skills for employment to youth with intellectual disability. Career Dev Transit Except Individ. (2020) 43(1):40–52. 10.1177/2165143418810671

[62] Plavnick JB Kaid T MacFarland MC. Effects of a school-based social skills training program for adolescents with autism spectrum disorder and intellectual disability. J Autism Dev Disord. (2015) 45(9):2674–90. 10.1007/s10803-015-2434-5 25820638

[63] Olsson NC Flygare O Coco C Görling A Råde A Chen Q Social skills training for children and adolescents with autism spectrum disorder: a randomized controlled trial. J Am Acad Child Adolesc Psychiatry. (2017) 56(7):585–92. 10.1016/j.jaac.2017.05.001 28647010

